# Early management of sepsis in medical patients in rural Thailand: a single-center prospective observational study

**DOI:** 10.1186/s40560-019-0407-z

**Published:** 2019-12-02

**Authors:** Kristina E. Rudd, Viriya Hantrakun, Ranjani Somayaji, Suchart Booraphun, Chaiyaporn Boonsri, Annette L. Fitzpatrick, Nicholas P. J. Day, Prapit Teparrukkul, Direk Limmathurotsakul, T. Eoin West

**Affiliations:** 1 International Respiratory and Severe Illness Center, University of Washington, Seattle, WA USA; 2 Division of Pulmonary, Critical Care, and Sleep Medicine, Department of Medicine, University of Washington, Seattle, WA USA; 3 Department of Critical Care Medicine, University of Pittsburgh, Pittsburgh, PA USA; 4 Mahidol-Oxford Tropical Medicine Research Unit, Faculty of Tropical Medicine, Mahidol University, Bangkok, 10400 Thailand; 5 Department of Medicine, The University of Calgary, Calgary, Alberta Canada; 6 Sunpasitthiprasong Hospital, Ubon Ratchathani, Thailand; 7 Departments of Family Medicine, Epidemiology, and Global Health, University of Washington, Seattle, WA USA; 8 Centre for Tropical Medicine and Global Health, Nuffield Department of Medicine, University of Oxford, Oxford, UK

**Keywords:** Sepsis, Critical care, Global health, Management, Thailand

## Abstract

**Background:**

The burden of sepsis is highest in low- and middle-income countries, though the management of sepsis in these settings is poorly characterized. Therefore, the objective of this study was to assess the early management of sepsis in Thailand.

**Methods:**

Pre-planned analysis of the Ubon-sepsis study, a single-center prospective cohort study of Thai adults admitted to the general medical wards and medical intensive care units (ICUs) of a regional referral hospital with community-acquired sepsis.

**Results:**

Between March 2013 and January 2017, 3,716 patients with sepsis were enrolled. The median age was 59 years (IQR 44-72, range 18-101), 58% were male, and 88% were transferred from other hospitals. Eighty-six percent of patients (N = 3,206) were evaluated in the Emergency Department (ED), where median length of stay was less than 1 hour. Within the first day of admission, most patients (83%, N = 3,089) were admitted to the general medical wards, while 17% were admitted to the ICUs. Patients admitted to the ICUs had similar age, gender, and comorbidities, but had more organ dysfunction and were more likely to receive measured sepsis management interventions. Overall, 84% (N = 3,136) had blood cultures ordered and 89% (N = 3,308) received antibiotics within the first day of hospital admission. Among the 3,089 patients admitted to the general medical wards, 38% (N = 1,165) received an adrenergic agent, and 21% (N = 650) received invasive mechanical ventilation. Overall mortality at 28 days was 21% (765/3,716), and 28-day mortality in patients admitted to the ICUs was higher than that in patients admitted to the general medical wards within the first day (42% [263/627] vs. 16% [502/3,089], p < 0.001).

**Conclusions:**

Sepsis in a regional referral hospital in rural Thailand, where some critical care resources are limited, is commonly managed on general medical wards despite high rates of respiratory failure and shock. Enhancing sepsis care in the ED and general wards, as well as improving access to ICUs, may be beneficial in reducing mortality.

**Trial registration:**

The Ubon-sepsis study was registered on clinicaltrials.gov (NCT02217592).

## Background

Sepsis, the dysregulated host response to infection that results in life-threatening organ dysfunction [1], is a global problem. Over 19 million cases of sepsis (formerly severe sepsis) and five million sepsis-related deaths are estimated to occur worldwide each year [2]. Although data are incomplete, the burden of sepsis is particularly high in resource-limited settings such as low- and middle-income countries (LMICs) [3]. It is therefore critical to better characterize sepsis in such environments.

Optimal emergency management of sepsis is evolving, and current evidence-based recommendations emphasize early sepsis recognition and initiation of antimicrobial therapies, and provision of necessary organ support [4]. While sepsis outcomes have been improving in recent years, perhaps due to increased clinical awareness [5, 6], the optimal approach to management remains unclear [7–10]. However, as most studies of sepsis management have been conducted in high-income countries, the applicability of these results to settings in low- or middle-income countries (LMICs) is uncertain.

There may be important differences in how and where patients with sepsis are managed in some LMIC settings. For example, Emergency Department (ED) capabilities and access to intensive care units (ICUs) may be restricted. Oxygen, antimicrobials, intravenous fluids, and vasoactive agents may not be available, or may be available in limited quantities. To better characterize sepsis care in a middle-income country setting, we analyzed the initial management of patients with community-acquired sepsis enrolled in the Ubon-sepsis study, a large prospective cohort study of Thai adults admitted to a tertiary referral hospital in rural Thailand [11].

## Methods

The Ubon-sepsis study was conducted in full compliance with the principles of good clinical practice and the ethical principles of the Declaration of Helsinki. The study protocol and related documents were approved by Sunpasitthiprasong Hospital Ethics Committee (039/2556), the Ethics Committee of the Faculty of Tropical Medicine of Mahidol University (MUTM2012-024-01), the University of Washington Division of Human Subjects (Institutional Review Board approval 42988), and the Oxford Tropical Research Ethics Committee of the University of Oxford (OXTREC172-12). The study is registered on clinicaltrials.gov (NCT02217592).

### Study design and study site

This is a pre-planned analysis of the Ubon-sepsis study, a prospective observational study of community-acquired sepsis at Sunpasitthiprasong Hospital, Ubon Ratchathani, Thailand, from March 2013 to January 2017 [11]. Sunpasitthiprasong Hospital is a public tertiary-care hospital with 1200 non-ICU beds and 220 ICU beds, serving a catchment area of 1.8 million people. Severely ill patients presenting to district hospitals are often referred to Sunpasitthiprasong Hospital, which is equipped with microbiology facilities and specialty physicians.

### Study participants

The study prospectively enrolled patients ≥ 18 years old who were admitted to the general medical wards or the medical ICUs with a primary diagnosis of infection made by the attending physician, were within 24 h of admission to the study hospital, and had at least three Surviving Sepsis Campaign criteria for sepsis documented in the medical record (Additional file 1: Table S1) [12]. We excluded patients who were diagnosed with hospital-acquired infections, had a previous hospitalization within the past 30 days, or were transferred from other hospitals with a total duration of hospitalization > 72 h. Patients under the age of 18 are admitted to separate pediatric wards and pediatric ICUs. Patients who have a primary diagnosis that is anticipated to require surgical intervention or who have undergone surgery during the present hospitalization are generally admitted to separate surgical wards and surgical ICUs.

Research nurses sequentially screened all medical patients by reviewing admission logs in the ED, medical wards, and medical ICUs. In addition, ED staff notified the study team directly about potentially eligible patients. Eligibility for the study was determined exclusively from data documented in the medical chart. For this analysis, we evaluated patients with sepsis, defined as infection plus the presence of organ dysfunction as per sepsis-3 guidelines [1]. Organ dysfunction was determined by a modified sequential (sepsis-related) organ failure assessment (SOFA) score, as previously described [11].

### Patient assessments

After enrollment, each patient was evaluated by the study team using four point-of-care assessments: a whole blood lactate rapid diagnostic test (RDT) (Lactate Pro 2, Arkray Global Business Inc., Australia), a whole blood glucose RDT (ACCU-CHECK Performa, Roche Diagnostic, Germany), pulse oximetry (Nellcor N-65, Covidien plc., Ireland), and Glasgow Coma Scale (GCS). The study did not involve any clinical interventions, and all treatment was provided by the medical teams. Mortality at 28 days after enrollment was evaluated via telephone contact if subjects were no longer hospitalized and had been discharged alive.

### Statistical analysis

Data were summarized with medians and interquartile ranges (IQR) for continuous measures, and proportions for discrete measures. IQR are presented in terms of 25th and 75th percentiles. Continuous variables and proportions were compared between groups using Mann-Whitney *U* tests and chi-square tests, respectively. Fisher’s exact test was used when any cell had a value less than 10. We assessed the association between clinical characteristics up to the time of study enrollment and admission to the ICUs using a multivariable logistic regression model. Covariates in the models, chosen a priori, included age, sex, presence of at least one comorbidity, pulse oximetry, SBP, GCS score, creatinine, bilirubin, platelets, presence of respiratory failure, receipt of adrenergic agent, transfer from ED or inpatient status at another medical facility, and nighttime admission. We performed a Kaplan-Meier analysis to assess 28-day survival by admitting location. All analyses were performed using Stata version 15.1 (StataCorp, College Station, TX, USA).

## Results

### Baseline characteristics

The Ubon-sepsis study enrolled 5001 patients from March 2013 to January 2017. Twelve patients were lost to follow-up and were excluded from analysis. Three thousand seven hundred and sixteen (74%) met the definition of sepsis at the time of study enrollment, exhibiting organ dysfunction as described above, and were retained for further study [11]. Median age was 59 years (IQR 44–72, range 18–101) and 2139 (58%) were male (Table 1). Half of patients (*N* = 1844) had a pre-existing medical condition, most commonly hypertension (*N* = 935, 25%). Up to the time of study enrollment, the median lowest recorded systolic blood pressure (SBP) was 90 mm Hg (IQR 73–103) and low oxygen saturation (SpO2 < 92%) was observed in 794 (21%) patients.

**Table 1:** Patient characteristics at enrollment

Variable	All patients (N = 3716)
Male, *n* (%)	2139 (58%)
Age (yrs), median (IQR)	59 (44–72)
Comorbidities, *n* (%)	1844 (50%)
Cancer	57 (2%)
Cerebrovascular disease	83 (2%)
Chronic kidney disease	515 (14%)
Diabetes mellitus	788 (21%)
Dyslipidemia	211 (6%)
Heart disease	224 (6%)
HIV	37 (1%)
Hypertension	935 (25%)
Liver disease	124 (3%)
Lung disease	292 (8%)
Lowest SpO_2_ (%), median (IQR; *N* = 3710) ^*a*^	97 (93-98)
Lowest SBP (mm Hg), median (IQR; *N* = 3713) ^*a*^	90 (73-103)
Lowest GCS ≤ 14, *n* (%; *N* = 3716) ^*a*^	689 (19%)
Highest creatinine (mg/dL), median (IQR; *N* = 3670) ^*a*^	1.6 (1.0–2.9)
Highest bilirubin (mg/dL), median (IQR; *N* = 2945) ^*a*^	0.8 (0.4–2.0)
Lowest platelets (cells/μL), median (IQR; *N* = 3706) ^*a*^	141,000 (67,000–228,000)

Measuring SpO2 and GCS at enrollment was part of the study protocol

*IQR* interquartile range, *HIV* human immunodeficiency virus, *SpO2* peripheral capillary oxygen saturation, *SBP* systolic blood pressure, *GCS* Glasgow Coma Scale

^*a*^Lowest SpO2, SBP, GCS, and platelets, and highest creatinine and bilirubin values up to the time of study enrollment are presented. *N* = total number of patients evaluated for those parameters up to the time of the study enrollment

### Prior emergency management

Of the 3716 patients with sepsis, 3264 (88%) were transferred to Sunpasitthiprasong Hospital from one of 63 referring hospitals. Of these patients, 807 (25%) had blood cultures ordered by their treating physicians and 2328 (72%) received at least one antibiotic prior to or during transfer. Ceftriaxone was the most commonly administered antibiotic (*N* = 1930, 59%). Most patients (*N* = 2607, 80%) received intravenous (IV) fluid at the referring facility or during transfer, and median fluid volume was 1200 mL (IQR 500–2000, range 100–7100). One thousand two hundred and twenty three (37%) patients received an IV adrenergic agent, most commonly dopamine, and 148 (5%) received mechanical ventilation.

### Emergency Department management

Of 3716 patients with sepsis, 3206 (86%) were first evaluated in the ED, and the remainder were first evaluated in the outpatient department. Of those evaluated in the ED, 2954 (92%) were transferred from other facilities (Additional file 1: Table S2 in the supplement). Among patients assessed in the ED, there was no significant difference between transferred and non-transferred patients in terms of age (*p* = 0.14) or sex (*p* = 0.84). However, transferred patients were generally sicker than non-transferred patients and were more likely to have at least one pre-existing comorbidity (*p* = 0.02). Median SBP measured in the ED for transferred patients was lower than that of non-transferred patients (110 mm Hg (IQR 95–130, range 42–251) vs. 117 mm Hg (IQR 96–141, range 56–232), *p* = 0.01).

Among the 3094 patients for whom hospital arrival time and ED departure time were available, the median ED length of stay was 30 min (IQR 20–45) for transferred patients and 45 min (IQR 25–75) for non-transferred patients. Of all 3206 patients evaluated in the ED, 2032 (63%) had blood cultures ordered and 2160 (67%) had at least one antibiotic prescribed. An additional 519 and 567 patients had blood cultures ordered and received antibiotics, respectively, prior to transfer. Therefore, a total of 2551 (80%) and 2724 (85%) patients had blood cultures and antibiotics, respectively, ordered by the end of their ED stay. Among the 2024 patients for whom hospital arrival time and antibiotic administration time at the study hospital were available, the median time from presentation to antibiotic administration was 3.2 h (IQR 1.6–5.8, range 0–32 h), indicating that most antibiotics were administered after patients left the ED. There was no difference in time to first antibiotic administration at the study hospital between patients who were or were not transferred from other facilities (*p* = 0.77). However, patients who were directly admitted to the ICUs had a shorter median time from presentation to antibiotic administration than those who were directly admitted to the general medical wards (2.3 h (IQR 1.4–4.1, *N* = 174) vs. 3.3 h (IQR 1.6–5.9, *N* = 1842), *p* = 0.002).

More transferred patients than non-transferred patients received IV crystalloid in the ED (64% (1891/2954) vs. 44% (112/252), *p* < 0.001). Among 98 patients with recorded IV fluid volume received in the ED, the median fluid volume was 500 mL (IQR 400–800, range 100–2500). More transferred patients than non-transferred patients received an adrenergic agent in the ED (42% (1253/2954) vs. 7% (18/252), *p* < 0.001); dopamine was prescribed most commonly. The proportion of patients who received mechanical ventilation in the ED was higher in transferred patients (26%, 781/2954) than in non-transferred patients (3%, 7/252; *p* < 0.001).

### Admission ward and management during the first hospital day

Of all 3716 patients, 297 (8%) were admitted directly to the ICUs. An additional 330 patients (9%) were transferred to the ICUs within the first hospital day, with a total of 627 patients (17%) who received ICU management within the first day of admission (Additional file 1: Table S3 in the supplement). Patients admitted to the ICUs during the first hospital day were more likely to receive all measured interventions, including antibiotics, blood cultures, mechanical ventilation, vasopressors, acute hemodialysis, and placement of a urinary catheter (Table 2; all *p* < 0.001). Nonetheless, one-third (38%; 1165/3089) of patients on the general medical wards received an adrenergic agent and many (21%; 650/3089) received invasive mechanical ventilation within the first day. Seventy-three percent of patients receiving adrenergic agents were admitted to the ward rather than the ICU (1165/1606), and more patients with respiratory failure requiring invasive mechanical ventilation were admitted to the ward than to the ICUs (57%; 650/1150).

**Table 2:** Clinical management within the first day of hospitalization

Therapy	All patients (N = 3716)	General ward (N = 3089)	ICU (N = 627)	*P* value
Antibiotic, *n* (%)	3308 (89%)	2703 (88%)	605 (96%)	< 0.001
Blood culture, *n* (%)	3136 (84%)	2573 (83%)	563 (90%)	< 0.001
Urinary catheterization, *n* (%)	2457 (66%)	1851 (60%)	606 (97%)	< 0.001
Acute dialysis, *n* (%)	33 (0.9%)	17 (0.6%)	16 (3%)	< 0.001
Adrenergic agent, *n* (%)	1606 (43%)	1165 (38%)	441 (70%)	< 0.001
Mechanical ventilation, *n* (%)	1150 (31%)	650 (21%)	500 (79%)	< 0.001

Management of general ward and ICU patients was compared using Mann-Whitney *U* test for continuous variables and chi-squared or exact tests for categorical variables

*ICU* intensive care unit

### Outcomes

**Fig. 1:**
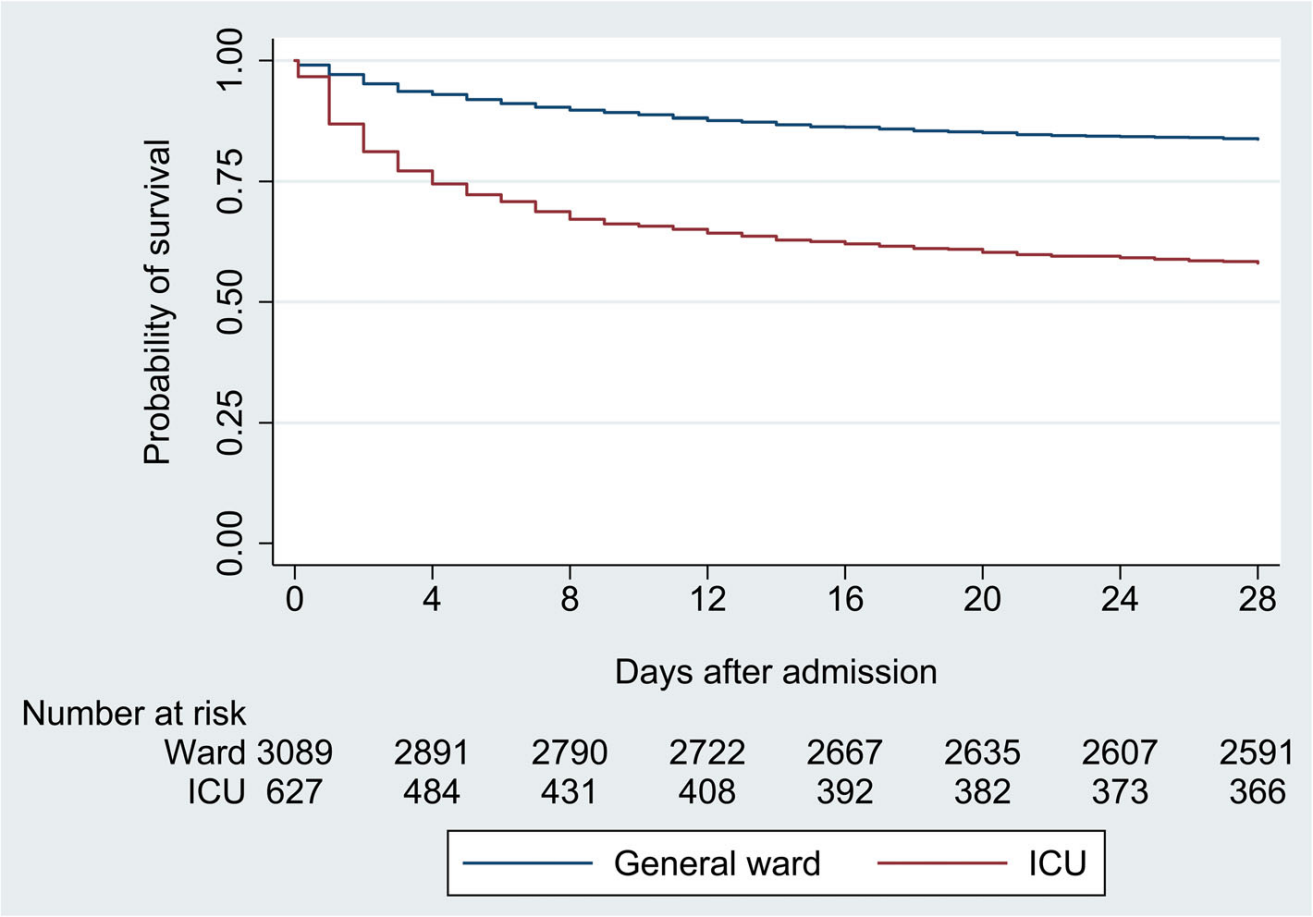
Kaplan-Meier curve demonstrating 28-day survival by admitting location

Overall mortality at 28 days was 21% (765/3716) and was higher for patients admitted to the ICUs (42%; 263/627) than for patients admitted to the general medical wards within the first hospital day (16%; 502/3089, *p* < 0.001). Survival curves demonstrated that the majority of deaths in patients admitted to the ICUs occurred within a week of admission (Fig. 1). Of 627 patients admitted to the ICUs within the first day of hospitalization, 28-day mortality did not differ between those patients who were directly admitted to the ICUs versus those who were initially admitted to the general medical wards and then transferred to the ICUs (hazard ratio, 1.11 [95% CI, 0.87–1.42], *p* = 0.38).

Overall hospital mortality was 8% (292/3716). The majority of patients who died after hospital discharge but within 28 days of enrollment were discharged in a moribund condition against medical advice (78%; 367/473). The median time to death for these 473 patients was less than 1 day after discharge (IQR 0–1, range 0–26).

### Clinical characteristics associated with ICU admission

The availability of ICU beds in Sunpasitthiprasong Hospital is far exceeded by the number of sepsis patients. To assess how access to this restricted resource is allocated, we performed univariable and multivariable analysis of patient characteristics up to the time of study enrollment associated with ICU admission (Additional file 1: Table S3, Table 3). Patients admitted to the ICUs were similar to those admitted to the general medical wards with respect to age, sex, and whether they were admitted during the day or night. However, patients admitted to the ICUs were more hypoxemic, more hypotensive, had lower GCS, and had higher plasma levels of creatinine and bilirubin (all *p* < 0.01). They were also more likely to have respiratory failure and to be receiving an adrenergic agent (both *p* < 0.001). In the multivariable model, younger age, lower SpO2, GCS ≤ 14, higher creatinine, respiratory failure, use of an adrenergic agent, and transfer from another hospital were significantly associated with admission to the ICUs within the first day after study enrollment (*p* < 0.05 for all).

**Table 3:** Association between patient characteristics at presentation and intensive care unit admission within the first hospital day

Variables	Number of Patients with Observation	Crude OR (95% CI)	*P* value	Adjusted OR (95% CI) ^*a*^	*P* value
Male sex	3716	1.13 (0.95–1.34)	0.18	0.91 (0.73–1.13)	0.38
Age (yrs)	3716	1.00 (0.99–1.00)	0.18	0.97 (0.96–0.98)	< 0.001
Presence of at least one comorbidity	3716	1.14 (0.96–1.35)	0.14	-	*-*
Lowest SpO_2_ (%)^*b*^	3710	0.94 (0.94–0.95)	< 0.001	0.98 (0.97–1.00)	0.005
Lowest SBP (mm Hg)^*b*^	3713	0.98 (0.98–99)	< 0.001	0.99 (0.98–0.99)	< 0.001
Lowest GCS ≤ 14^*b*^	3716	3.35 (2.77–4.05)	< 0.001	1.29 (1.00–1.67)	0.05
Highest creatinine (mg/dL)^*b*^	3670	1.08 (1.05–1.11)	< 0.001	1.08 (1.05–1.12)	< 0.001
Highest bilirubin (mg/dL)^*b*^	2945	1.04 (1.02–1.07)	0.002	-	-
Lowest platelets (cells/μL)^*b*^	3706	1.00 (1.00–1.00)	0.98	-	-
Required mechanical ventilation	3716	11.9 (9.77–14.6)	< 0.001	15.3 (11.7–19.9)	< 0.001
Received adrenergic agent	3716	3.41 (2.84–4.09)	< 0.001	3.41 (2.61–4.45)	< 0.001
Transferred from other hospitals	3716	5.80 (3.59–9.36)	< 0.001	1.97 (1.14–3.41)	0.02
Night time admission	3569	0.95 (0.79–1.13)	0.54	-	-

*OR* odds ratio, *CI* confidence interval, *SpO2* peripheral capillary oxygen saturation, *SBP* systolic blood pressure, *GCS* Glasgow Coma Scale

^*a*^Using multivariable logistic regression (*N* = 3661)

^*b*^Lowest SpO2, SBP, GCS, and platelets; highest creatinine and bilirubin values; and clinical management up to the time of study enrollment

## Discussion

This prospective observational study of adults with community-acquired sepsis at a regional Thai referral hospital provides important new data about patient characteristics and early management in this setting. The most not able findings are that while the ED stay was remarkably short, most patients had antibiotics prescribed and blood cultures ordered in the ED; the antibiotics were largely administered later, when patients were admitted to the general medical wards or the ICUs. In addition, the large majority of community-acquired sepsis patients were admitted to general medical wards rather than to the ICUs (83% vs. 17%) within the first hospital day. Those who were admitted to the ICUs were sicker and had higher mortality. While mechanical ventilation and adrenergic agents were implemented frequently on both the general medical wards and in the ICU, initiation of hemodialysis was uncommon. Sepsis in this setting is a frequently fatal condition.

While there are several well-equipped ICUs in the study hospital, during the study period these were frequently near capacity. As a result, critically ill patients were often admitted to the general medical wards. Indeed, during the first day of hospitalization more than half of patients with respiratory failure or shock were managed outside of the ICUs. Strategies to reduce mortality from sepsis in this and other LMIC settings may thus need to include improving access to ICUs or high dependency units, as well as promoting research and professional guidelines on sepsis care outside of the ICU when ICUs are not available or are fully occupied.

Over 80% of medical ward patients and 90% of ICU patients received at least one antibiotic within the first day of hospitalization. All patients presenting with sepsis should receive early antibiotics; this treatment difference between ICU and non-ICU settings perhaps reflects a lower patient-to-provider ratio in the ICUs. Further assessment of the determinants of this difference could inform future sepsis management guidelines and implementation. Over 80% of patients in this study had blood cultures ordered by attending physicians within the first day of admission. This is significantly higher than reported in other observational studies of sepsis patients in LMICs. Conde et al. reported that, among patients in 19 different hospitals in Brazil, only 16.3% of patients in public hospitals and 34.1% of patients in private hospitals had blood cultures performed [13]. The high percentage of blood culture testing in this study is also supported by an increasingly high total number of blood culture bottles utilized at the study hospital each year [14].

It is notable that so many patients had blood cultures ordered and antibiotics prescribed during their time in the ED, despite the fact that median ED length of stay was less than an hour for both transferred and non-transferred patients. Median time patients with sepsis spend in the ED reflects local practice, and may differ widely by location. However, many studies have reported significantly longer median times to antibiotics in the ED for septic patients, including those performed in high resource settings, and the time from presentation the ED until antibiotic administration has been consistently shown to be related to mortality in sepsis [15–17].

While overall hospital mortality was low at 8%, some patients were discharged in a moribund condition and, due to local preference, were sent home with the expectation that they would die shortly after discharge. Thus, in this study, 28-day mortality may be the more robust mortality measurement and the best point of comparison with other studies. The 28-day mortality of 21% overall and 42% for patients admitted to the ICUs was similar to or lower than that reported in several other LMICs. For example, Silva et al. reported 47.3% 28-day mortality for adults admitted to ICUs with severe sepsis in Brazil, and Andrews et al. reported 45% 28-day mortality for hospitalized adults with sepsis in the standard-of-care arm of a randomized clinical trial in Zambia [10, 18]. The 28-day mortality in this study is also similar to the in-hospital mortality reported in several studies of patients in high-resource settings. Rhee et al. reported 15% in-hospital mortality and 6.2% discharge to hospice among adult patients with sepsis in 409 U.S. hospitals in 2014, and there was 18.9% in-hospital mortality among patients in the standard-of-care arm of the ProCESS trial [9, 19]. This comparable mortality with higher resource settings despite limited resources and different management practices could be due to several contributing factors, such as different causative pathogens [20] or different characteristics of the study population. Additionally, it is possible that the mortality in this study is spuriously low, as the very high proportion of transferred patients introduces the potential for survivorship bias. Further studies are needed to assess the impact of different sepsis management strategies in diverse settings, including in smaller district hospitals.

This study has several limitations. First, we are unlikely to have enrolled all sepsis patients presenting during the study period. Although study staff consecutively screened all patients admitted to the general medical wards and ICUs, screening was based upon a clinical diagnosis of suspected infection listed in the patient’s chart, and some patients with sepsis may have been initially misdiagnosed with non-infectious conditions. Additionally, surgical, obstetric, and pediatric patients were not enrolled. Second, unique characteristics of this study population, such as low HIV prevalence and a very high proportion of patients who were transferred from other facilities, may limit external validity. Third, as an observational study, most of the data were limited to those routinely collected by clinicians in the study hospital. Fourth, as this was a single-center study, our findings may not reflect the management of patients with sepsis elsewhere in Thailand.

There are several strengths of this study. The size—larger than most studies of sepsis management and outcomes in LMICs to date—is a major asset. Patients were consecutively enrolled, limiting potential bias. The follow-up rate was excellent, with only 12 of 5001 patients (0.2%) missing vital status at 28 days. Following patients to 28 days after enrollment allowed us to capture a more robust measure of case fatality in this population. Additionally, as 88% of patients were transferred to the study hospital from one of 63 hospitals, we were able to assess early patient characteristics and management at these referring centers.

## Conclusions

In summary, this prospective observational study provides essential data about the characteristics, early management, and outcomes of patients with sepsis at a large public Thai hospital, most of them were referred from over 60 smaller hospitals. While the sickest patients were managed in the ICUs, the large majority of patients were managed on the general medical wards, including those with respiratory failure or shock. This study is among the largest published prospective analyses of patients with sepsis in a LMIC, and suggests that enhancing sepsis care in the ED and general medicals wards, as well as improving access to ICUs, may be beneficial in reducing sepsis mortality.

## Supplementary information

**Additional file 1: Table S1.** Patients required at least 3 of the following modified Surviving Sepsis Campaign diagnostic criteria for sepsis for study enrollment. **Table S2.** Emergency Department management of Thai adult patients with community-acquired sepsis. **Table S3.** Patient characteristics within the first hospital day, by hospital location.

## Data Availability

The datasets used during the current study are available from the corresponding author on reasonable request.

## Abbreviations

ED: Emergency Department
ICU: Intensive care unit
LMICs: Low- and middle-income countries
RDT: Rapid diagnostic test (RDT)
GCS: Glasgow Coma Scale
IQR: Interquartile range
SBP: Systolic blood pressure
SpO2: Peripheral capillary oxygen saturation
IV: Intravenous
HIV: Human Immunodeficiency Virus
OR: Odds ratio
CI: Confidence interval
