# Supplementary material for: Early management of sepsis in medical patients in rural Thailand: a single-center prospective observational study
Source: J Intensive Care. 2019 Dec 2;7:55. doi: 10.1186/s40560-019-0407-z (PMC6886203; doi:10.1186/s40560-019-0407-z)
Supplement: Supplementary file 1 — Additional file 1: Table S1. Patients required at least 3 of the following modified Surviving Sepsis Campaign diagnostic criteria for sepsis for study enrollment. Table S2. Emergency Department management of Thai adult patients with community-acquired sepsis. Table S3. Patient characteristics within the first hospital day, by hospital location. [file 40560_2019_407_MOESM1_ESM.docx]

**Additional file 1**

**Table S1. Patients required at least 3 of the following modified Surviving Sepsis Campaign diagnostic criteria for sepsis for study enrollment**

| **Infection, documented or suspected, and some of the following:** |
| --- |
| **General variables** |
| Fever or hypothermia (body temperature >38.3^o^C or <36^o^C) *^a^* |
| Heart rate >90/min |
| Tachypnea (respiratory rate >20/min) *^b^* |
| Altered mental status (Glasgow Coma Scale <15) *^c^* |
| Significant edema or positive fluid balance (>20 mL/kg over 24 hr) |
| Hyperglycemia (plasma glucose >140 mg/dL) in the absence of diabetes |
| **Inflammatory variables** |
| Leukocytosis (WBC count >12,000 u/L), Leukopenia (WBC count <4,000 u/L), or immature forms >10% *^d^* |
| Plasma C-reactive protein >2 SD above the normal value |
| Plasma procalcitonin >2 SD above the normal value |
| **Hemodynamic variables** |
| Arterial hypotension (SBP <90 mmHg, MAP <70 mmHg or an SBP decrease >40 mmHg) |
| **Organ dysfunction variables** |
| Low oxygen saturation determined by pulse oximetry (SpO_2_ <95%) *^e^* |
| Arterial hypoxemia (PaO_2_/FiO_2_ <300) |
| Acute oliguria (urine output <0.5 mL/kg/hr for at least 2 hrs) *^f^* |
| Creatinine increase >0.5 mg/dL |
| Coagulation abnormalities (INR >1.5 or aPTT >60 s) |
| Ileus (absent bowel sounds) |
| Thrombocytopenia (platelet count <100,000 u/L) |
| Hyperbilirubinemia (plasma total bilirubin >4 mg/dL) |
| **Tissue perfusion variables** |
| Hyperlactatemia (>1 mmol/L) |
| Decreased capillary refill or mottling |

Abbreviations: WBC, white blood cell; SBP, systolic blood pressure; MAP, mean arterial pressure; SpO2, peripheral capillary oxygen saturation; PaO_2_, arterial partial pressure of oxygen; FiO_2_, fraction of inspired oxygen; INR, international normalized ratio; aPTT, activated partial thromboplastin time.

Adapted from Dellinger et al, Surviving Sepsis Campaign: International Guideline for Management of Severe Sepsis and Septic Shock, 2012 [12]

*^a^* Fever and hypothermia were consolidated into a single variable.

*^b^* Respiratory rate >20/min was defined for the tachypnea variable.

*^c^* Glasgow Coma Scale ≤14 was defined for the altered mental status variable.

*^d^* Leukocytosis, Leukopenia, and immature forms >10% were consolidated into a single variable.

*^e^* Low oxygen saturation determined by pulse oximetry (SpO_2_ <95%) was added.

*^f^* The condition of ‘despite adequate fluid resuscitation’ for this criterion was omitted.

**Table S2. Emergency Department management of Thai adult patients with community-acquired sepsis**

| **Therapy** | **All Patients**  **Treated in ED**  **N = 3206** | **Transferred Patients**  **Treated in ED**  **N = 2954** | **Non-Transferred Patients**  **Treated in ED**  **N = 252** | **P-value** |
| --- | --- | --- | --- | --- |
| Antibiotic, *n* (%) *^a^* | 2157 (67%) | 1999 (68%) | 161 (64%) | 0.22 |
| Ceftriaxone | 1408 (44%) | 1287 (44%) | 121 (48%) | 0.17 |
| Ceftazidime | 526 (16%) | 503 (17%) | 23 (10%) | **0.001** |
| Clarithromycin | 110 (3%) | 95 (3%) | 15 (6%) | **0.02** |
| Clindamycin | 315 (10%) | 300 (10%) | 15 (6%) | **0.03** |
| Doxycycline | 240 (7%) | 227 (8%) | 13 (5%) | 0.14 |
| Metronidazole | 177 (6%) | 172 (6%) | 5 (2%) | **0.009** |
| IV crystalloid, *n* (%) | 2003 (62%) | 1891 (64%) | 112 (44%) | **<0.001** |
| Blood culture, *n* (%) | 2032 (63%) | 1840 (62%) | 192 (76%) | **<0.001** |
| Arterial blood gas, *n* (%) | 248 (8%) | 240 (8%) | 8 (3%) | **0.004** |
| Adrenergic agent, *n* (%) | 1271 (40%) | 1253 (42%) | 18 (7%) | **<0.001** |
| Mechanical ventilation, *n* (%) | 788 (25%) | 781 (26%) | 7 (3%) | **<0.001** |

Management of transfer and non-transfer patients was compared using Mann-Whitney U test for continuous variables and Chi-squared or Exact tests for categorical variables.

*^a^* Six most commonly prescribed antibiotics are listed

**Table S3. Patient characteristics within the first hospital day, by hospital location**

| **Variable** | **General ward**  **N=3,089** | **ICU**  **N=627** | **P-value** |
| --- | --- | --- | --- |
| Male, *n* (%) | 1,763 (57%) | 376 (60%) | 0.18 |
| Age (yrs), median (IQR) | 60 (44-73) | 57 (44-70) | 0.06 |
| Co-morbidities, *n* (%) | 1,516 (49%) | 328 (52%) | 0.14 |
| Cancer | 55 (2%) | 2 (0.3%) | **0.004** |
| Cerebrovascular disease | 67 (2%) | 16 (3%) | 0.56 |
| Chronic kidney disease | 442 (14%) | 73 (12%) | 0.08 |
| Diabetes mellitus | 615 (20%) | 173 (28%) | **<0.001** |
| Dyslipidemia | 178 (6%) | 33 (5%) | 0.62 |
| Heart disease | 183 (6%) | 41 (7%) | 0.56 |
| HIV | 33 (1%) | 4 (0.6%) | 0.51 |
| Hypertension | 781 (25%) | 154 (25%) | 0.70 |
| Liver disease | 103 (3%) | 21 (3%) | 0.99 |
| Lung disease | 240 (8%) | 52 (8%) | 0.66 |
| Lowest SpO_2_ (%), median (IQR) *^a^* | 97 (94-99) | 94 (85-98) | **<0.001** |
| Lowest SBP (mm Hg), median (IQR) *^a^* | 90 (77-106) | 80 (70-98) | **<0.001** |
| Lowest GCS≤14, *n* (%) *^a^* | 458 (15%) | 231 (37%) | **<0.001** |
| Highest creatinine (mg/dL), median (IQR) *^a^* | 1.5 (1.0-2.7) | 2.2 (1.4-3.7) | **<0.001** |
| Highest bilirubin (mg/dL), median (IQR) *^a^* | 0.8 (0.4-1.9) | 1.1 (0.5-2.6) | **<0.001** |
| Lowest platelets (cells/μL), median (IQR) *^a^* | 141,000 (68,000-226,000) | 140,000 (59,000-233,000) | 0.59 |
| Venous lactate (mmol/L), median (IQR) | 1.9 (1.3-2.8) | 3.4 (2.0-7.1) | **<0.001** |
| Modified SOFA score, median (IQR) *^b^* | 4 (3-6) | 7 (5-11) | **<0.001** |
| Required mechanical ventilation, *n* (%) *^a^* | 619 (20%) | 470 (75%) | **<0.001** |
| Received adrenergic agents, *n* (%) *^a^* | 1,152 (37%) | 420 (67%) | **<0.001** |
| Transferred from another facility, *n* (%) *^a^* | 2,637 (86%) | 609 (97%) | **<0.001** |
| Initial presentation to the emergency department, *n* (%) | 2,593 (84%) | 613 (98%) | **<0.001** |
| Night time admission, *n* (%) | 1,155 (39%) | 230 (38%) | 0.54 |

Characteristics of patients admitted to the general medical ward and ICU were compared using Mann-Whitney U tests for continuous variables and Chi-squared tests for categorical variables.

*^a^* Lowest SpO2, SBP, GCS, and platelets; highest creatinine and bilirubin values; and clinical management up to the time of the study enrollment are presented

*^b^* As previously described, the SOFA score was modified for this study [11]. The study did not record inotropic and vasopressor agent doses, and PaO2/FiO2 indices were not available for the majority of patients due to the infrequency of arterial blood gas (ABG) testing. Therefore, the cardiovascular component of the SOFA score was modified such that patients received a component score of 2 (on a 4-point scale) if they received only dobutamine or dopamine, and scored a component score of 3 if they received epinephrine or norepinephrine. The respiratory component of the SOFA score was modified such that patients received a component score of 2 (on a 4-point scale) if they received invasive mechanical ventilation and an ABG was not performed.
